# Endoscopic submucosal dissection with deliberate muscularis propria exposure for curative resection of a large type 1 gastric neuroendocrine tumor: a case report

**DOI:** 10.3389/fonc.2026.1872571

**Published:** 2026-07-01

**Authors:** Nana An, Xiuli Zheng, Limian Er, Lei Zhang

**Affiliations:** Department of Endoscopy, The Fourth Hospital of Hebei Medical University, Shijiazhuang, China

**Keywords:** gastric neuroendocrine tumor, endoscopic submucosal dissection, endoscopic ultrasound, multidisciplinary team, endoscopic intermuscular dissection

## Abstract

**Background:**

The management of large (>2 cm) type 1 gastric neuroendocrine tumors (gNETs), typically associated with autoimmune gastritis, remains controversial, balancing curative intent against procedural invasiveness. This report aims to demonstrate that, with precise preoperative staging and advanced endoscopic techniques, curative organ-preserving resection is a viable option for selected cases.

**Case presentation:**

A 48-year-old female presented with markedly elevated serum gastrin (3100 pg/mL) and gastroscopy revealed two dominant lesions: a 2.5 cm lesion at the esophagogastric junction and a 1.8 cm lesion on the gastric body, the background gastric mucosa showed atrophy with scattered nodules. Comprehensive staging with endoscopic ultrasound and 68Ga-DOTATATE PET/CT, incorporating histopathology from biopsy confirming a type I gNET, G2 grade, established a localized, non-metastatic disease. After multidisciplinary discussion, the patient opted for endoscopic resection. The junctional tumor was completely removed en bloc using a tailored technique of endoscopic submucosal dissection with deliberate muscularis propria exposure (ESD-MPE), while the gastric body lesion was resected via conventional ESD. Final pathology confirmed both as pT1b, G2 NETs with all margins negative.

**Conclusion:**

For carefully selected, larger type I gNETs without metastasis, ESD−MPE represents an effective minimally invasive treatment option. This technique reflects the trend toward deeper and more precise endoscopic therapy.

## Introduction

Gastric neuroendocrine tumors (gNETs) derive from enterochromaffin-like cells and are increasing in incidence (1). The 2019 WHO classification grades them as G1-G3 based on the Ki-67 index for prognosis (2), while the Rindi system classifies them into types 1–3 to guide management (3). Type I gastric NETs are typically associated with autoimmune gastritis and hypergastrinemia, usually presenting as multiple, low-grade submucosal lesions (4).

For type I gNETs larger than 2 cm, the choice between endoscopic resection and surgical intervention remains controversial (5–7). This report describes a case of a 2.5 cm G2 grade type I gNET with markedly elevated gastrin levels (3100 pg/mL). After precise staging with endoscopic ultrasound (EUS) and 68Ga-DOTATATE PET/CT ruled out metastasis, the lesion was successfully resected en bloc using endoscopic submucosal dissection with deliberate muscularis propria exposure (ESD-MPE). This case demonstrates that, with careful patient selection based on multimodal imaging, ESD-MPE can provide a curative minimally invasive treatment for select larger type 1 gNETs, offering a new therapeutic perspective for this clinical gray area.

## Case report

**Figure 1:**
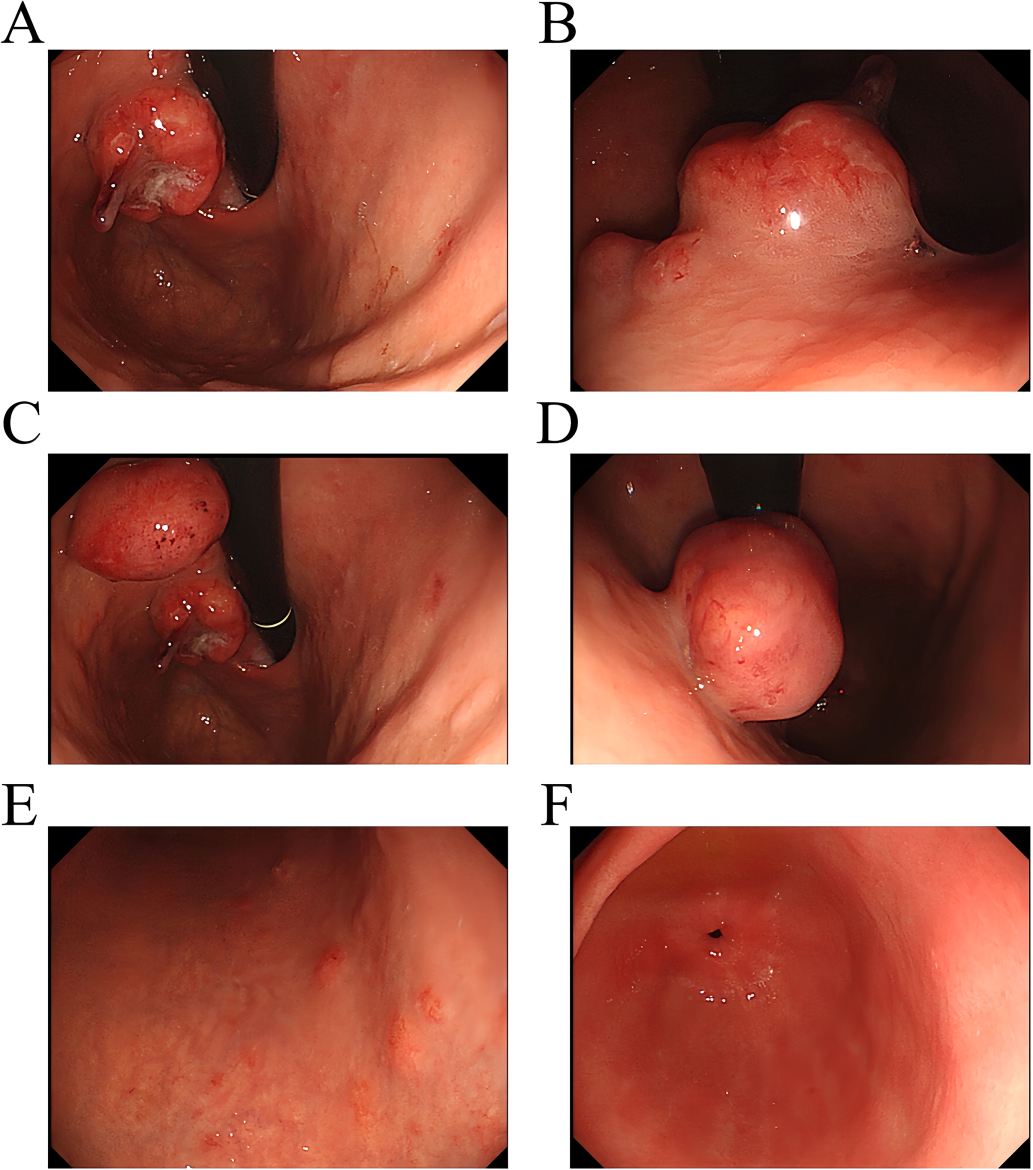
Initial gastroscopic findings. **(A, B)** A protruding lesion approximately 2.5 cm in diameter is seen at the esophagogastric junction, with a central depression and surface covered by mucus. **(C, D)** A subpedunculated, protruding lesion approximately 1.5 cm in size is observed on the mid-posterior wall of the gastric body. **(E)** Scattered, slightly elevated nodules are present in the gastric body, accompanied by thinning of the mucosa exhibiting atrophic changes. **(F)** The antral mucosa appears smooth, erythematous, and shows no signs of atrophy.

On November 27, 2024, a 48-year-old female underwent gastroscopy during a health checkup, which revealed a 2.5 cm broad-based elevated lesion with central ulceration and hyperemia located at the esophagogastric junction, along with a 1.5 cm pedunculated polyp on the mid-posterior gastric wall (**Figures 1A–D**). The patient had no prior history of PPI use and no history of Helicobacter pylori eradication. The carbon-14 urea breath test was positive at 230 dpm (normal <100 dpm), indicating mild infection. Serum gastrin was markedly elevated at 3100 pg/mL (**Table 1**). Gastrointestinal endoscopy also revealed atrophic changes extending from the gastric body to the fundus, while the antrum appeared normal (**Figures 1E, F**). Based on these endoscopic features, differential diagnoses included hyperplastic polyps, inflammatory polyps, and neoplasms with malignant potential. Histopathological examination, however, confirmed a grade G2 gastric NET. Notably, in the presented case, the markedly elevated serum gastrin level (3100 pg/mL), the endoscopic findings of thinning gastric body mucosa with atrophic changes, and the presence of multiple scattered nodules are highly characteristic of a type I gastric neuroendocrine tumor. The patient was ultimately diagnosed with a type I gastric neuroendocrine tumor, G2 grade. The patient reported occasional reflux symptoms, with no other significant complaints or notable medical history.

**Table 1:** Results of laboratory investigations on the first visit.

Test items	Test results	Reference interval	Unit
White blood cell (WBC)	5.56	3.5-9.5	× 10^9^/L
Red blood cellss (RBC)	4.47	4.3-5.8	× 10^9^/L
Hemoglobin	145	130-175	g/L
Hematocrit	0.434	0.4-0.5	
Mean corpuscular volume (MCV)	97.1	82-100	fL
Platelet count	298	125-350	× 10^9^/L
Alanine aminotransferase (ALT)	34.2	9-50	U/L
Aspartate aminotransferase (AST)	21.4	15-40	U/L
Albumin	46.8	40-55	g/L
Na	138	135-150	mmol/L
K	4.2	3.5-5.5	mmol/L
Gastrin	3100	0-100	Pg/mL
*Helicobacter pylori*	230	0-100	dpm

**Figure 2:**
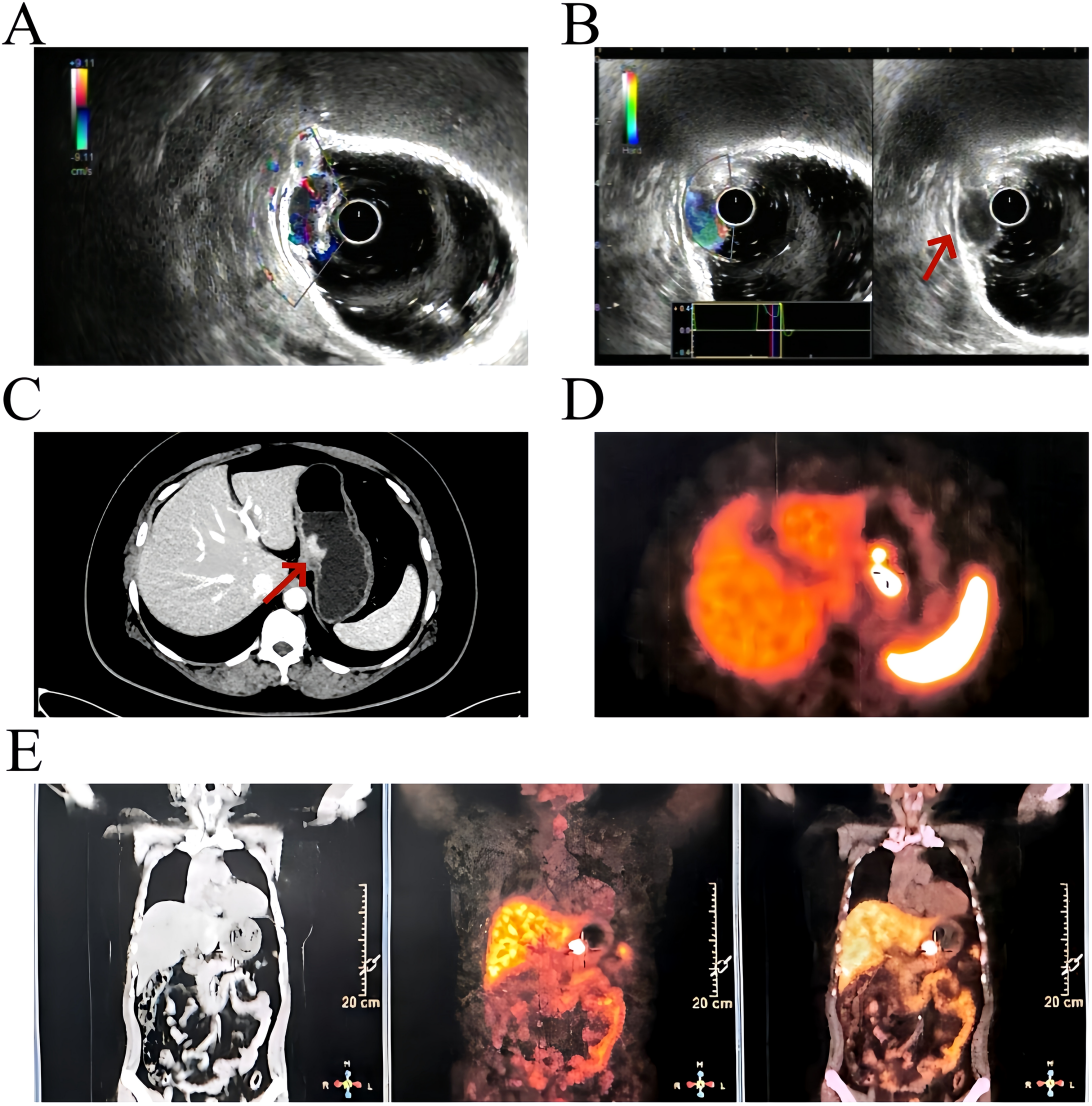
Diagnostic imaging. **(A, B)** Endoscopic ultrasound revealed two distinct lesions: a hypoechoic mass measuring 23.3 × 16.9 mm, located at the esophagogastric junction, involving the mucosal and submucosal layers (red arrow) with relatively abundant blood flow signals and firm consistency; and a separate homogeneous polypoid lesion in the gastric body confined to the mucosa. **(C)** Abdominal CT shows an intraluminal protruding mass at the esophagogastric junction (red arrow), which demonstrates marked heterogeneous enhancement in the arterial phase and homogeneous enhancement in the portal venous phase on contrast-enhanced scan. **(D, E)** 68Ga−DOTATATE PET/CT reveals wall thickening and an intraluminal protruding mass at the esophagogastric junction, with corresponding abnormal radiotracer avidity on PET. No significant abnormal metabolic activity is observed elsewhere in the body.

Endoscopic ultrasound revealed two distinct lesions: a hypoechoic mass measuring 23.3 × 16.9 mm, located at the esophagogastric junction, involving the mucosal and submucosal layers with relatively abundant blood flow signals and firm consistency; and a separate homogeneous polypoid lesion in the gastric body confined to the mucosa (**Figures 2A, B**). Abdominal CT shows an intraluminal protruding mass at the esophagogastric junction, which demonstrates marked heterogeneous enhancement in the arterial phase and homogeneous enhancement in the portal venous phase on contrast-enhanced scan (**Figure 2C**). 68Ga−DOTATATE PET/CT reveals wall thickening and an intraluminal protruding mass at the esophagogastric junction, with corresponding abnormal radiotracer avidity on PET. No significant abnormal metabolic activity is observed elsewhere in the body (**Figures 2D, E**). Based on the diagnostic findings, the patient was assigned a clinical stage of T1bN0M0.

A multidisciplinary team (MDT) consisting of gastroenterologists, gastrointestinal surgeons and medical oncologists comprehensively reviewed the patient’s laboratory, endoscopic, imaging and preliminary pathological data. Three therapeutic strategies were proposed with reference to relevant consensus guidelines after evaluating technical feasibility, curative efficacy and long-term prognosis:

1. Non-surgical management: High-dose proton pump inhibitors (PPIs) or somatostatin analogues (SSAs) were administered to suppress excessive gastric acid secretion, with PPIs recommended as first-line agents to prevent peptic complications. This regimen was not suitable for curative treatment, as two large submucosal invasive grade 2 lesions carried risks of local progression.
2. Surgical interventions: (1) Laparoscopic wedge resection, proximal gastrectomy or total gastrectomy; (2) Combined laparoscopic-endoscopic resection. Surgery failed to eradicate diffuse enterochromaffin-like (ECL) cell hyperplasia, which necessitated lifelong endoscopic surveillance after operation. In addition, surgical resection would lead to irreversible gastric dysfunction, including refractory reflux, dumping syndrome and malabsorption.
3. Endoscopic resection: The procedure was individualized according to EUS and PET/CT findings. Endoscopic submucosal dissection with muscularis propria exposure (ESD-MPE) was performed for the lesion at the esophagogastric junction to achieve en bloc resection, while standard ESD was adopted for the gastric body lesion. Both techniques aimed at complete tumor resection while maintaining the integrity of the muscular layer.

The patient was staged T1bN0M0, with lesions confined to the submucosa and no lymph node or distant metastasis, conferring an extremely low metastatic risk. Our center has accumulated ample experience in ESD-MPE for lesions at the esophagogastric junction, allowing complete R0 resection with comparable radical efficacy to laparoscopic wedge resection. This approach eliminated high-risk lesions while preserving native gastric function.

The definitive treatment plan would be adjusted according to pathological margin status; salvage surgery would be indicated if deep resection margins tested positive. Endoscopic resection offered minimal trauma and superior postoperative quality of life, despite a potential risk of incomplete resection. By contrast, surgical resection provided reliable radical clearance yet entailed greater operative trauma and persistent long-term gastric dysfunction. After thorough guideline-based discussion of risks and benefits, the patient elected to receive endoscopic resection.

## 1 Preoperative ethical communication

The patient was informed of the higher perforation risk compared to conventional ESD, alternative surgical options (laparoscopic wedge resection, subtotal or total gastrectomy), and their associated long-term risks. We explained that ESD-MPE could achieve R0 resection while preserving gastric integrity. All potential adverse events, including perforation, bleeding, and possible emergency surgery, were disclosed. After full discussion, the patient voluntarily provided written informed consent.

## 2 Preoperative risk assessment

Preoperative EUS confirmed the 2.5 cm EGJ lesion had only superficial submucosal invasion without deep muscular involvement or transmural fibrosis. CT and ^68^Ga-DOTATATE PET/CT excluded serosal involvement and extragastric extension. The MDT assessed the EGJ anatomy: given its thin-walled nature and rich vascularity, careful dissection along the muscularis propria plane was planned, with hemostatic forceps used to pretreat traversing vessels. In the event of uncontrollable bleeding or perforation, conversion to surgery remains an option.

## 3 Procedure steps:

**Figure 3:**
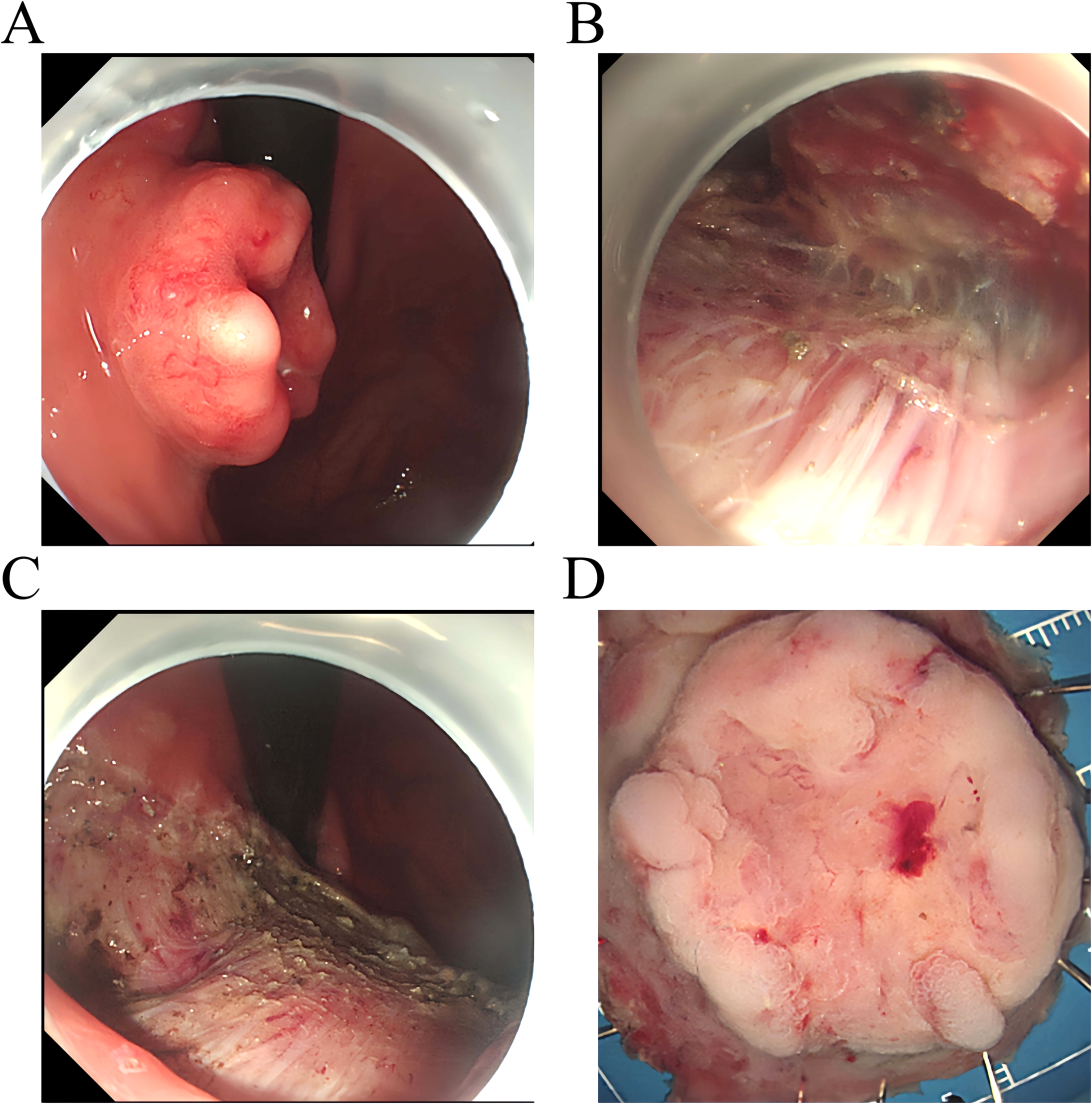
Tumor resection at the esophagogastric junction using the ESD-MPE technique. **(A)** A protruding tumor measuring approximately 2.5cm located at the esophagogastric junction, with a central depression. **(B)** Dissection was performed along the plane of the muscularis propria using a mucosal incision knife, with careful attention to avoid injury to the muscle layer; vessels traversing the submucosal and muscular layers were prophylactically coagulated. **(C)** The wound bed after complete en bloc resection of the tumor, showing full exposure of the intact muscularis propria without evidence of bleeding or perforation due to muscular injury. **(D)** Fixation of the resected specimen.

1. Operator qualifications: The procedure was performed by an endoscopist with over 8 years of experience in ESD and a long-standing practice in treating lesions at the esophagogastric junction.
2. Surgical planning: Given the expected procedure and anesthesia duration, resection prioritized the two larger tumors: one at the esophagogastric junction (**Figure 3A**) and the other on the mid-posterior gastric wall.
3. Marking and submucosal injection: Circumferential markings were made with a mucosal incision knife (Micro-Tech, Nanjing), followed by submucosal injection of methylene blue-saline solution to lift the lesions.
4. Circumferential incision and dissection:
  1. Tumor of the esophagogastric junction (using ESD-MPE technique): Using a mucosal incision knife, the incision was carefully deepened until the glistening muscularis propria was fully exposed. Submucosal dissection then proceeded meticulously along this plane (**Figure 3B**), with maintained traction and counter-traction to facilitate layer separation and minimize perforation risk. Vessels traversing the submucosa and muscle layer were prophylactically coagulated to prevent retraction-related bleeding.
  2. Tumor of the gastric body: Conventional ESD was performed.
5. Wound and specimen management:

The wound was inspected, and hemostasis was achieved by coagulating visible vessels. Muscularis propria integrity was confirmed (**Figure 3C**). Specimens were fixed (**Figure 3D**) and submitted for pathological evaluation of lateral and deep margins, along with maximum invasion depth.

## 4 Postoperative pathological findings

### 1) Tumor of the esophagogastric junction

Size: 2.5 cm × 2.2 cm × 1.2 cm. Neuroendocrine tumor, G2, invading the submucosa (depth ~1000 μm). Pathological Stage: pT1b (according to AJCC 8th edition). No definite lymphovascular or perineural invasion. Immunohistochemistry: AE1/AE3(+), CgA(+), CD56(+), Syn(+), Ki-67 (~4% in hotspots), CEA(–), CDX2(–/+), Desmin (muscle+). Margins: Oral, anal, lesser curvature, greater curvature, and deep margins – all negative.

### 2) Tumor of the gastric body

Size: 1.8 cm × 1.5 cm × 1.5 cm. Neuroendocrine tumor, G2, involving submucosa (depth ~300 μm). Pathological Stage: pT1b (according to AJCC 8th edition). No definite lymphovascular or perineural invasion. Immunohistochemistry: AE1/AE3(+), CgA(+), CD56(+), Syn(+), Ki-67 (~5% in hotspots), CEA(–), CDX2(–/+), Desmin (muscle+). Margins: Oral, anal, lesser curvature, greater curvature, and deep margins – all negative.

## 5 Postoperative course

The procedure was completed in 120 minutes without intraoperative complications. The patient received prophylactic cefazolin and remained afebrile with no signs of infection. A liquid diet was started on postoperative day (POD) 3, advanced to semi-liquid on POD 5 before discharge, and soft solid food by POD 7. At the 2-month follow-up, endoscopy showed a well-healed scar. The patient was readmitted for endoscopic mucosal resection of the remaining small neuroendocrine tumor and subsequently received somatostatin analog therapy. Subsequent follow-up was performed at 6 months and 12 months. The most recent follow-up was in January 2026, which showed no evidence of recurrence or metastasis. Thereafter, annual surveillance was recommended (**Table 2**).

**Table 2:** Clinical timeline flowchart.

Time point	Clinical event	Sequential arrow
January 2026	Most recent surveillance endoscopy; no local recurrence or distant metastasis; annual endoscopic surveillance recommended	←
July 2025	6-month postoperative routine endoscopic follow-up	←
February 2025	2-month postoperative surveillance gastroscopy showing complete wound healing; readmission for EMR of residual tiny neuroendocrine lesion; somatostatin analog therapy initiated	←
02 December 2024	Curative endoscopic procedure: ESD-MPE for EGJ lesion + standard ESD for gastric body lesion, uneventful operation	←
01 December 2024	Multidisciplinary team (MDT) discussion; three treatment regimens compared; patient consented to endoscopic resection	←
27 November 2024	Health check gastroscopy detected two gastric lesions; blood tests, EUS, abdominal CT and ^68^Ga-DOTATATE PET/CT completed; confirmed diagnosis of type I G2 gNET cT1bN0M0	Starting point

The study was carried out after the protocol was approved by the Ethics Committee of the Fourth Hospital of Hebei Medical University. The patient gave written informed consent for the publication of clinical details and images.

## Discussion

This report presents a case of a type I gNET, G2 grade, approximately 2.5 cm in diameter. After preoperative imaging excluded lymph node or distant metastasis, the lesion was successfully removed en bloc using ESD−MPE. This case suggests that for carefully selected larger type I gNET, ESD−MPE may serve as an effective minimally invasive treatment option.

Management of type I gNET requires an individualized approach based on tumor size, grade, depth of invasion, and metastatic risk (8). For lesions of type I gNETs larger than 1 cm in diameter or with elevated Ki-67 expression, endoscopic resection should be considered (9, 10). Somatostatin analogs can achieve high response rates but are generally used as long−term therapy due to frequent recurrence after discontinuation (11). Evidence supports the use of endoscopic resection as the preferred treatment for lesions ≤2 cm confined to the mucosa or submucosa (12, 13). However, management of tumors >2 cm remains controversial: some recommend surgical resection with lymph node dissection (5); meanwhile, multiple clinical investigations indicate that endoscopic resection can be safely performed in carefully screened low-risk individuals following complete preoperative staging workup (6, 7, 14). Notably, in the absence of clear surgical indications—such as lymph node metastasis or deep invasion—no conclusive evidence currently supports or opposes surgical intervention based solely on the presence of necrosis, lymphovascular invasion, or an elevated Ki-67 index (15, 16). Accurate preoperative staging, particularly assessment of lymph node and distant metastasis, is essential for decision−making.

Precise imaging is essential for planning local resection. Endoscopic ultrasound (EUS) provides clear visualization of tumor invasion depth and regional lymph nodes, playing a key role in assessing resectability and selecting appropriate candidates for endoscopic treatment (5). In addition, ^68^Ga-DOTATATE PET/CT offers high sensitivity and specificity for detecting occult primary lesions, staging, and evaluating liver metastases (17, 18), thereby providing reliable complementary information for treatment planning. Together, these modalities help define the extent of disease and support individualized decision-making.

The ESD-MPE technique emphasizes complete exposure of the muscularis propria to facilitate en bloc resection of submucosal lesions while preserving the integrity of the muscular layer. This approach is conceptually similar to endoscopic intermuscular dissection (EID), which has been described for rectal neuroendocrine tumors (19, 20), by enabling dissection along well-defined anatomical planes, ESD-MPE allows for accurate pathological assessment and helps guide further management strategies, including the need for additional treatment or surveillance.

In routine clinical practice, larger type I gastric NETs exceeding 2 cm in diameter are often empirically considered unsuitable for endoscopic resection based on conventional clinical reasoning, and consequently EUS may not be pursued. Moreover, larger NETs frequently demonstrate deep submucosal invasion or infiltration of the muscularis propria on endoscopic ultrasound, raising concerns about the feasibility of achieving complete resection and the potential need for salvage surgery following attempted endoscopic removal. Additionally, the relatively high cost of ^68^Ga-DOTATATE PET/CT may limit its accessibility for many patients, thereby reducing the number of candidates eligible for precise preoperative staging. The present case is unique in that both EUS and PET/CT were performed and yielded concordant findings: EUS confirmed only superficial submucosal invasion, and PET/CT excluded lymph node and distant metastasis. Despite the tumor size exceeding 2 cm, this favorable staging profile rendered endoscopic radical resection feasible, and the patient fully consented to this approach.

The patient was satisfied with the endoscopic treatment, as it avoided open surgery and enabled faster recovery. Although a second procedure was needed for a residual tumor, she found it acceptable. She tolerated the somatostatin analog therapy well and appreciated the clear communication from the care team.

Innovations of this study: Firstly, to our knowledge, this is the first reported application of the ESD-MPE technique for type I gastric NETs >2 cm, providing a preliminary reference for endoscopic management of lesions in this therapeutic gray zone where the role of endoscopic resection remains uncertain. Secondly, this case highlights the critical role of precise preoperative staging, particularly the combined use of EUS and ^68^Ga-DOTATATE PET/CT, in identifying suitable candidates for endoscopic radical resection.

This study also has several limitations that must be acknowledged. (1) The observations are derived from an individual patient case and require validation through larger-scale studies to confirm their generalizability. (2) ESD−MPE has a steep learning curve and requires substantial operator skill and institutional expertise. Importantly, ESD-MPE is not a routine therapy for large type I gNETs; it is only suitable for rigorously staged low-risk T1bN0 lesions. (3) No dedicated preoperative biopsies of background atrophic oxyntic mucosa or normal antral mucosa were performed, lacking preoperative histopathological confirmation of autoimmune atrophic gastritis and ECL cell hyperplasia, though this was partially compensated by peritumoral atrophic mucosa in ESD-MPE specimens. (4) Furthermore, follow−up duration is short, and long−term outcomes and recurrence risk require further observation. Prospective multicenter studies are needed to clarify the role, long−term efficacy, and optimal indications of ESD−MPE for larger type I gNETs.

In conclusion, for carefully selected, larger type I gNETs without metastasis, ESD−MPE represents an effective minimally invasive treatment option. This technique reflects the trend toward deeper and more precise endoscopic therapy.

## Data Availability

The original contributions presented in the study are included in the article/Supplementary Material. Further inquiries can be directed to the corresponding author.
